# Serological and molecular detection and phylogenetic characterization of Taylorella equigenitalis in horses in Iraq

**DOI:** 10.14202/vetworld.2026.4244-4258

**Published:** 2026-09-24

**Authors:** Zaid Salah Hussein, Amanee Mohammed Radhy, Saba Thamer Mosa, Hasanain A. J. Gharban

**Affiliations:** 1 Zoonoses Research Unit, College of Veterinary Medicine, University of Baghdad, Baghdad, Iraq.; 2 Department of Internal and Preventive Veterinary Medicine, College of Veterinary Medicine, University of Baghdad, Baghdad, Iraq.; 3 Department of Internal and Preventive Veterinary Medicine, College of Veterinary Medicine, University of Wasit, Wasit, Iraq.

**Keywords:** contagious equine metritis, equine reproductive disease, horses, Iraq, molecular detection, phylogenetic analysis, seroprevalence, Taylorella equigenitalis

## Abstract

**Background and Aim:**

Contagious equine metritis caused by *Taylorella* *equigenitalis* is an important venereal disease of equids that can impair reproductive performance and facilitate transmission through clinically inapparent carriers. Information on *T. equigenitalis* infection in Iraqi horses remains limited. This study aimed to determine the serological and molecular occurrence of *T. equigenitalis* in horses in Iraq, evaluate associations with clinical and epidemiological factors, and characterize PCR-positive isolates using sequencing and phylogenetic analysis.

**Materials and Methods:**

A total of 157 adult horses from Baghdad, Babylon, Nineveh, and Wasit provinces were examined from March to September 2025. Clinical information was recorded, and blood and genital swab samples were collected. Serum samples were examined using a qualitative enzyme-linked immunosorbent assay (ELISA), whereas DNA extracted from genital swabs was subjected to conventional polymerase chain reaction (PCR) targeting the *16S rRNA* gene. PCR-positive products were sequenced, submitted to GenBank, and analyzed phylogenetically. Associations between infection status and clinical and epidemiological variables were statistically evaluated.

**Results:**

Of the 157 horses, 52 (33.12%) were ELISA-positive and 23 (14.65%) were PCR-positive for *T. equigenitalis*. Twenty-one (13.38%) horses were positive by both methods, 31 (19.74%) were ELISA-positive/PCR-negative, two (1.27%) were ELISA-negative/PCR-positive, and 103 (65.61%) were negative by both methods. In mares, seropositivity was significantly associated with reproductive disorders, with the highest proportion detected among those with abnormal vaginal discharge (68.42%), whereas PCR positivity was highest among mares with infertility (50%). Seropositivity was higher in horses aged >10 years (62.86%) and females (52.38%), whereas PCR positivity was highest among horses aged >5–6 years (38.1%) and females (25%). Sequencing of 23 PCR-positive isolates generated GenBank accession numbers PZ146151.1–PZ146173.1. The Iraqi isolates showed 97.18%–99.12% sequence identity with an Iranian *T. equigenitalis* isolate (DQ840456.1).

**Conclusion:**

The findings provide serological and molecular evidence of *T. equigenitalis* infection among horses in Iraq and demonstrate associations with reproductive and epidemiological characteristics. Combined serological and molecular surveillance, routine screening of breeding horses, and higher-resolution molecular studies are warranted to clarify transmission dynamics and strengthen prevention and control strategies.

## INTRODUCTION

*Taylorella equigenitalis* is a highly specialized bacterial pathogen of equids and the etiological agent of contagious equine metritis (CEM). The disease was first recognized in 1977 in Newmarket, England, and was subsequently reported in the United States in 1978 and in numerous countries across multiple continents [1]. The organism was initially classified as *Haemophilus equigenitalis* based on its phenotypic characteristics; however, subsequent biochemical and molecular genetic analyses resulted in its reclassification into the genus *Taylorella*, family Alcaligenaceae, order Burkholderiales [2, 3]. The genus *Taylorella* currently comprises two recognized species: *T. equigenitalis*, which is primarily pathogenic in horses (*Equus caballus*), and *Taylorella asinigenitalis*, which is primarily associated with donkeys (*Equus asinus*) and is generally considered non-pathogenic [4, 5].

Venereal transmission through natural mating represents the principal route of *T. equigenitalis* transmission. The bacterium can colonize specific anatomical sites of the external genitalia of stallions, which may subsequently transmit the organism to the reproductive tract of mares during breeding [6, 7]. Experimental studies have demonstrated efficient venereal transmission, with clinical infection developing in a substantial proportion of mares bred to infected stallions [8, 9]. Iatrogenic transmission through contaminated reproductive or veterinary equipment represents another potential route of disease spread. Transmission from infected mares to foals has also been reported; however, the epidemiological significance and mechanisms of this transmission remain incompletely understood, and some exposed animals may subsequently become carriers [10, 11].

Clinical manifestations of *T. equigenitalis* infection occur predominantly in mares and may include abnormalities of the estrous cycle, temporary infertility, endometritis, cervicitis, vaginitis, and salpingitis [12–14]. The severity of clinical manifestations can vary considerably, ranging from subclinical infection to pronounced acute reproductive disease. In contrast, infected stallions are generally asymptomatic and may act as clinically inapparent carriers, creating an important challenge for disease detection and transmission control [12–14].

Isolation and culture of *T. equigenitalis* from genital swabs have historically served as important methods for confirming CEM; however, bacteriological diagnosis requires specialized culture conditions, and the organism is slow-growing and may require additional confirmatory testing [9]. Several serological approaches, including the complement fixation test (CFT), immunofluorescence assays, and enzyme-linked immunosorbent assay (ELISA), have also been used to detect antibodies against *T. equigenitalis*. Nevertheless, serological testing has limitations, including an inability to reliably distinguish current from previous exposure and variability in the timing and magnitude of antibody responses among individual animals [15–17]. Molecular methods, particularly polymerase chain reaction (PCR) assays targeting the 16S ribosomal RNA (*16S rRNA*) gene, provide an additional approach for detecting *T. equigenitalis* [18–20]. Sequencing and phylogenetic analysis of PCR products can further support molecular characterization and enable comparison of detected sequences with reference sequences deposited in international nucleotide databases [20, 21].

Despite the reproductive and epidemiological importance of CEM, information regarding *T. equigenitalis* infection in horses in Iraq remains limited. In particular, the available literature does not establish previous Iraqi data describing the serological or molecular occurrence of *T. equigenitalis*, its association with reproductive disorders and epidemiological characteristics, or the genetic relationships of locally detected sequences with strains reported elsewhere. This lack of integrated serological, molecular, epidemiological, and phylogenetic information represents an important knowledge gap because clinically inapparent carriers and horses with reproductive abnormalities may facilitate unrecognized maintenance or dissemination of the organism. Generating baseline evidence on the occurrence and molecular characteristics of *T. equigenitalis* is therefore important for understanding its potential relevance to equine reproductive health in Iraq.

Therefore, this study aimed to investigate *T. equigenitalis* infection in horses in Iraq using complementary serological and molecular approaches. Specifically, the study aimed to screen horses for antibodies against *T. equigenitalis* using ELISA; detect *T. equigenitalis* DNA using PCR; evaluate associations between infection status and reproductive disorders and selected epidemiological factors, including age, sex, and geographic region; and characterize PCR-positive sequences through sequencing and phylogenetic analysis to determine their genetic relationships with reference sequences deposited in the National Center for Biotechnology Information (NCBI) GenBank database. Collectively, these approaches were intended to provide baseline epidemiological and molecular information on *T. equigenitalis* infection in Iraqi horses.

## MATERIALS AND METHODS

### Ethical approval

The study protocol was reviewed and approved by the Central Scientific Committee, College of Veterinary Medicine, University of Wasit (Approval No. CSC-CVM-71; January 25, 2025), and the Institutional Animal Care and Use Committee, College of Veterinary Medicine, University of Wasit (Approval No. WU-CVM.CVM-IPM-1085; February 14, 2025). All procedures involving the study horses, including clinical examination and the collection of blood and genital swab samples, were conducted in accordance with the approved study protocol and institutional animal care requirements.

### Study period and location

The study was conducted from March to September 2025 in selected areas of four Iraqi provinces: Baghdad, Babylon, Nineveh, and Wasit. Baghdad, the capital of Iraq, is located in central Iraq at approximately 33°20′ N, 44°26′ E. Babylon is located in central Iraq, approximately 85 km south of Baghdad, at 32°32′ N, 44°25′ E. Nineveh is located in northern Iraq, approximately 410 km north of Baghdad, at 36°21′ N, 43°09′ E. Wasit is located in eastern Iraq, southeast of Baghdad, at approximately 32°30′ N, 45°49′ E. The study areas were selected principally according to the availability of horses and the cooperation and logistical support provided by horse owners and veterinary clinics during sample collection. The geographic distribution of the sampled horses across the four provinces is presented in Figure 1. 

**Figure 1:**
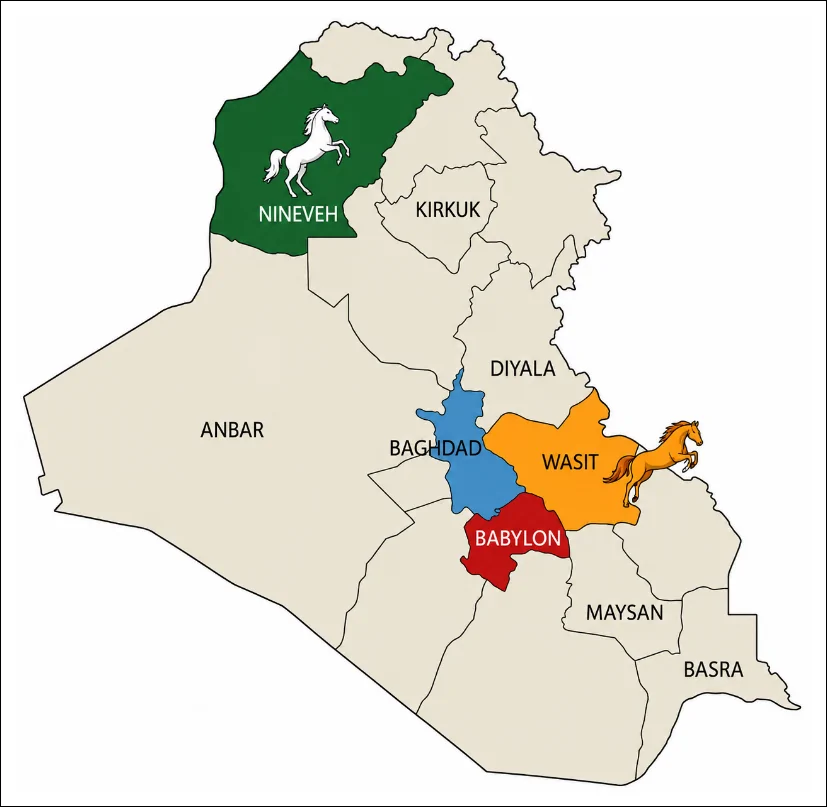
Geographic distribution of horses included in the study across the selected Iraqi provinces of Baghdad, Babylon, Nineveh, and Wasit.

### Study design

This study used a cross-sectional design to investigate the serological and molecular occurrence of *T. equigenitalis* and its associations with reproductive and selected epidemiological characteristics in horses from four Iraqi provinces. A total of 157 adult horses aged ≥4 years, with or without a history of reproductive disorders, were included. Clinical information was recorded for each horse before blood and genital swab samples were collected. Serum samples were examined for antibodies against *T. equigenitalis* using ELISA, whereas genital swab samples were examined for *T. equigenitalis* DNA using PCR. PCR-positive products were subsequently sequenced and subjected to phylogenetic analysis. The resulting serological and molecular findings were evaluated in relation to clinical and epidemiological variables. These procedures are consistent with the study population and diagnostic workflow reported in the manuscript.

### Study animals and sample collection

A total of 157 adult horses aged ≥4 years, with or without a history of reproductive disorders, were included in the study. Following clinical examination, approximately 5 mL of jugular venous blood was collected aseptically from each horse into a labeled anticoagulant-free gel tube. In addition, genital swab samples were obtained from the vagina of mares and from the urethral fossa, urethra, and prepuce of stallions. Blood and swab samples were transported to the laboratory under cooled conditions. Blood samples were centrifuged at 3000 × *g* for 5 min, after which the separated serum was transferred into labeled 1.5-mL microcentrifuge tubes. Serum and genital swab samples were stored at −40°C until serological and molecular examination, respectively.

### Serology

Serum samples were examined using a qualitative Horse *Taylorella equigenitalis* ELISA Kit (Cat. No. SL0011Ho; SunLong Biotech, China) according to the manufacturer’s instructions. Kit reagents and serum samples were brought to room temperature (22–27°C) before analysis. After completion of the assay procedures, absorbance was measured at 450 nm and expressed as optical density (OD). Following assay validation using the positive and negative controls, the critical cutoff value was calculated according to the manufacturer’s instruct-tions, and samples were classified as positive or negative based on their respective OD values.

### Molecular testing

DNA was extracted from genital swab samples using the Type A protocol of the G-Spin™ Total DNA Extraction Kit (iNtRON Biotechnology, Korea) according to the manufacturer’s instructions. DNA purity and concentration were evaluated using a NanoDrop spectrophotometer (Thermo Fisher Scientific, USA), with reported purity values of 1.8–2.0 and concentrations of 50–150 ng/µL. PCR targeted the *16S rRNA* gene using the primers TEF (5′-AATACCGCATACGCCCTGAG-3′) and TER (5′-GTATTATCCCAGGGGGCTGC-3′), which were designed based on the *T. equigenitalis* reference sequence deposited in NCBI GenBank (accession No. NR_029234.1).

DNA amplification was performed using AccuPower® PCR PreMix (Bioneer, Korea). Each reaction had a final volume of 25 µL comprising 7 µL of DNA template, 2 µL of forward primer, 2 µL of reverse primer, and 14 µL of nuclease-free water. Amplification was performed in a thermal cycler (Bio-Rad, USA) under the following conditions: initial denaturation at 95°C for 5 min; 35 cycles of denaturation at 95°C for 40 s, annealing at 56°C for 40 s, and extension at 72°C for 40 s; followed by final extension at 72°C for 5 min.

PCR products were separated by electrophoresis on 1.5% agarose gels stained with ethidium bromide at 100 V and 80 mA for 60 min. Standard *T. equigenitalis* DNA purchased from Macrogen (Korea) was used as the positive control, whereas nuclease-free water was used as the negative control. Amplified products were visualized using an ultraviolet transilluminator, and samples producing the expected 583-bp amplicon were considered PCR-positive.

### Phylogenetic analysis

PCR-positive products for *T. equigenitalis* were sequenced by Macrogen (Korea) using a modified Sanger sequencing method. The resulting sequences were aligned using ClustalW implemented in MEGA version 11 and subsequently submitted to the NCBI GenBank database to obtain accession numbers. Multiple sequence alignment, phylogenetic tree construction, sequence identity, nucleotide diversity (π), mean genetic distance, and evolutionary divergence between groups were evaluated using MEGA version 11 and DnaSP version 5.1. Haplotype analysis was also performed using the sequence data. The manuscript subsequently reports that 23 PCR-positive sequences received GenBank accession numbers PZ146151.1–PZ146173.1.

### Statistical analysis

Data were entered into Microsoft Excel 2016 and statistically analyzed using GraphPad Prism version 8.0.2. Associations between categorical variables were evaluated using Pearson’s chi-square (χ²) test or Fisher’s exact test, as appropriate. The Wilson method was used to calculate 95% confidence intervals (95% CI). Odds ratios (ORs), relative risks (RRs), and their corresponding 95% CIs were calculated using MedCalc Statistical Software. Data were expressed as number and percentage [n (%)] or mean ± standard error (SE), as appropriate. Differences were considered statistically significant at p < 0.05.

## RESULTS

### Serological findings

ELISA detected antibodies against *T. equigenitalis* in 52 of 157 horses (33.12%), whereas 105 (66.88%) were seronegative (Figure 2). The mean OD value of ELISA-positive samples (0.386 ± 0.009) was significantly higher than that of ELISA-negative samples (0.184 ± 0.003) (Figure 3).

**Figure 2:**
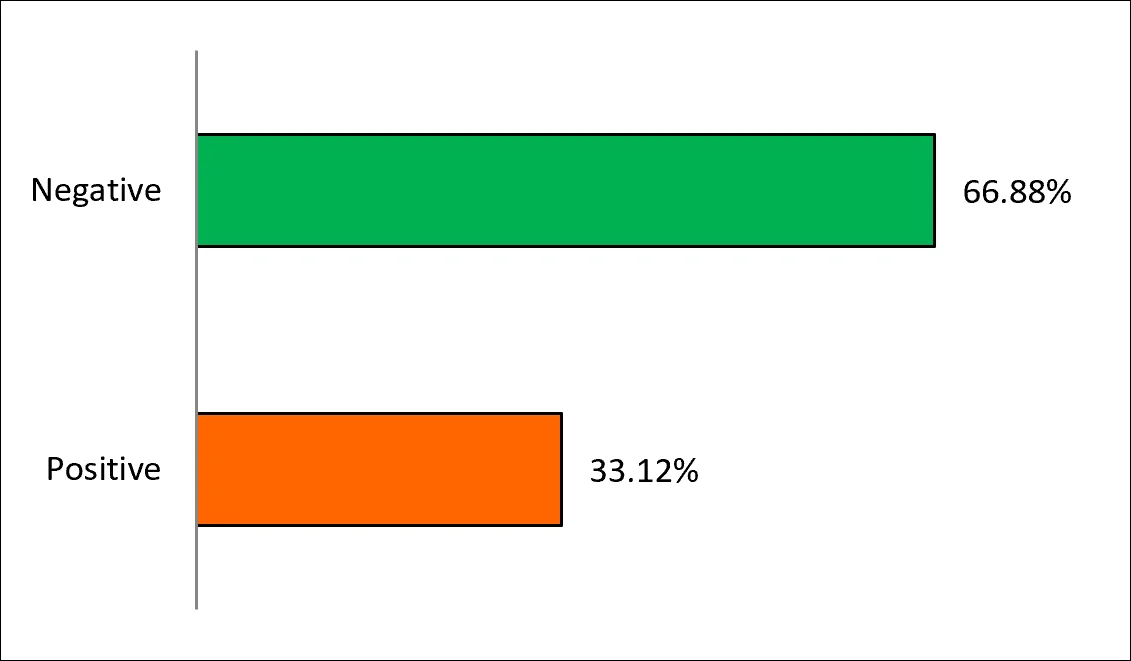
Serological detection of *Taylorella* *equigenitalis* antibodies by ELISA among the 157 study horses.

**Figure 3:**
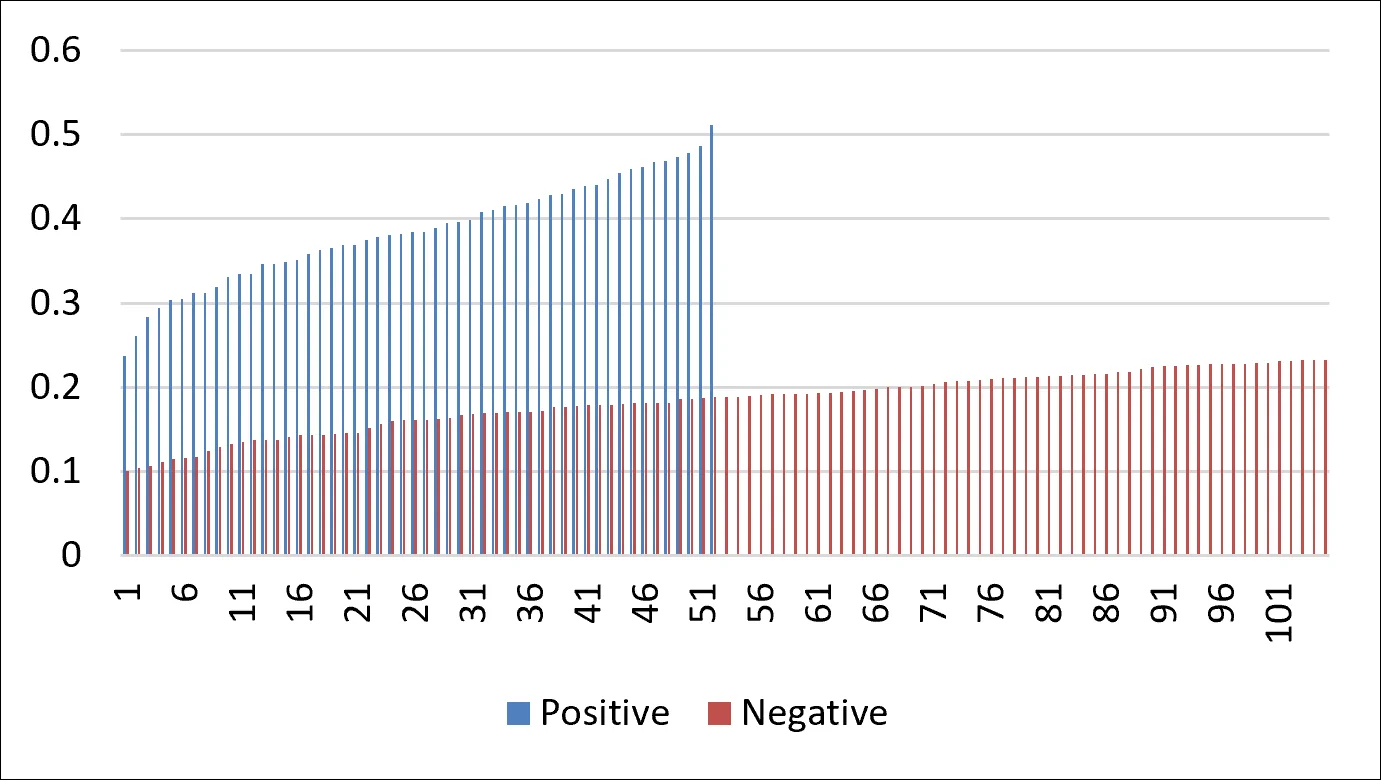
OD values of ELISA-positive and ELISA-negative serum samples. The cutoff OD value was 0.23315; samples with OD values ≥0.23315 were classified as positive for antibodies against *Taylorella* *equigenitalis*, whereas those with OD values <0.23315 were classified as negative.

### Molecular findings

PCR analysis of genital swab samples detected *T. equigenitalis* DNA in 23 of 157 horses (14.65%), whereas 134 (85.35%) were PCR-negative (Figure 4).

**Figure 4:**
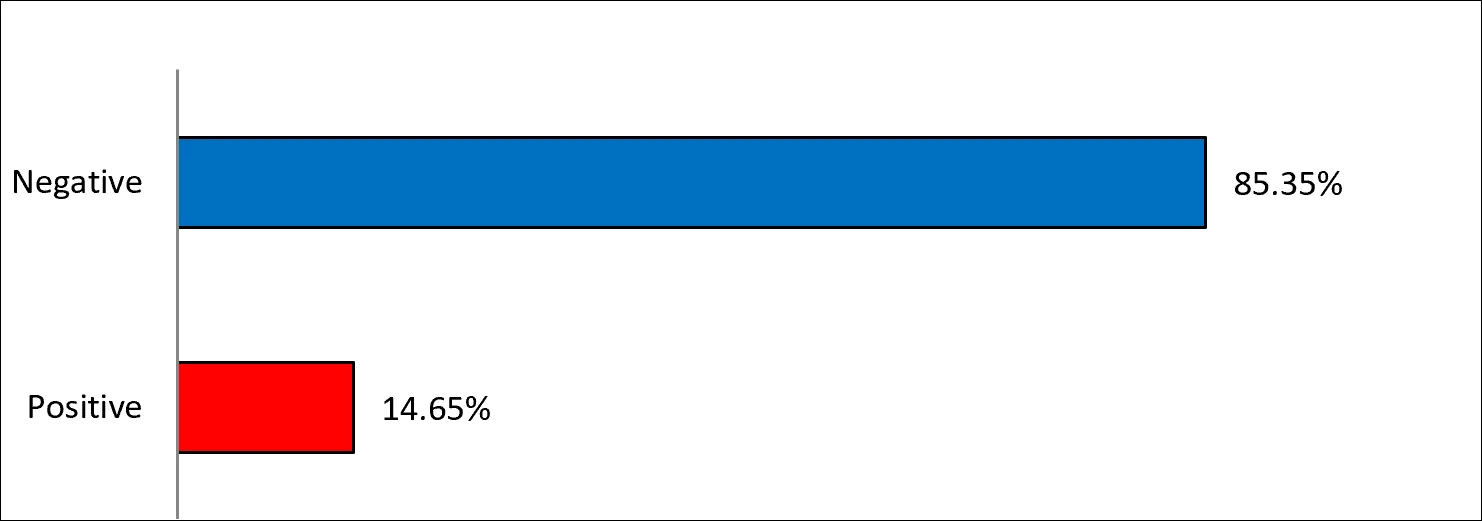
Molecular detection of *Taylorella* *equigenitalis* by polymerase chain reaction among the 157 study horses.

### Comparison of ELISA and PCR findings

Among the 157 examined horses, 21 (13.38%) were positive by both ELISA and PCR, 31 (19.74%) were ELISA-positive/PCR-negative, two (1.27%) were ELISA-negative/PCR-positive, and 103 (65.61%) were negative by both methods (Figure 5). These distributions are consistent with the overall ELISA and PCR positivity reported for the study population.

**Figure 5:**
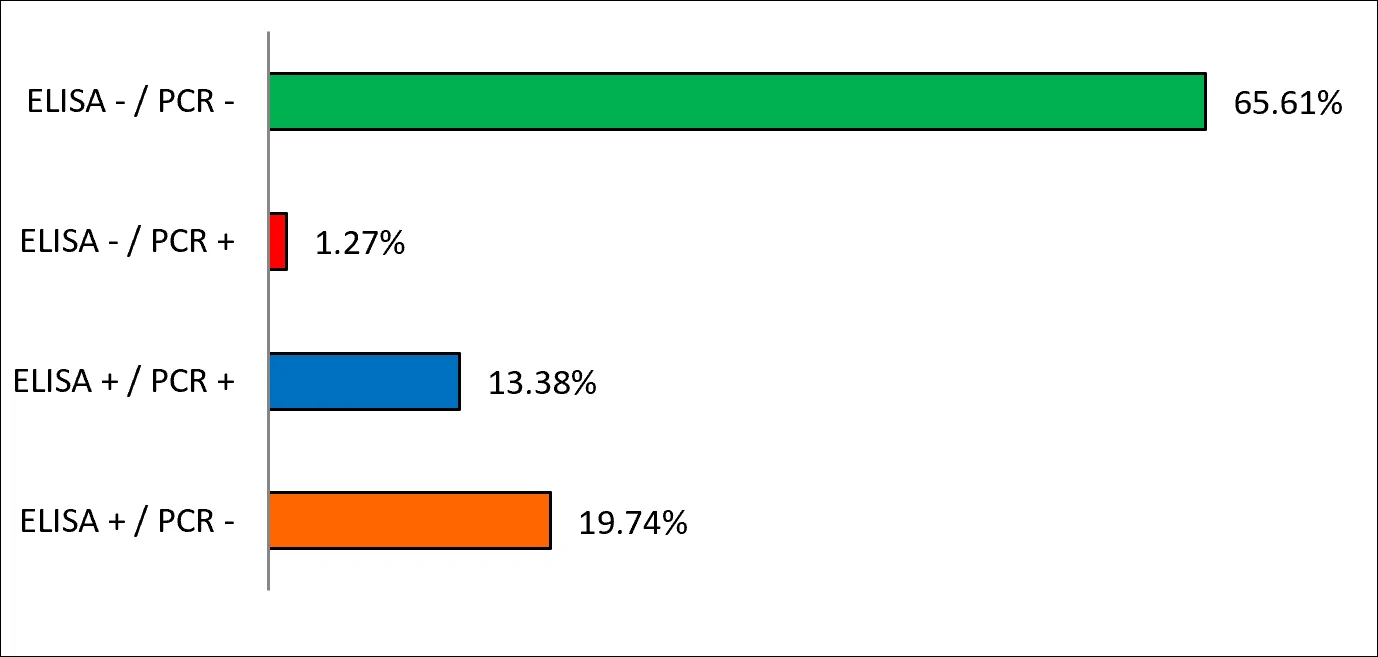
Distribution of combined enzyme-linked immunosorbent assay and polymerase chain reaction results among the study horses.

### Association of seropositivity with clinical findings

Among mares, ELISA seropositivity differed significantly according to reproductive and clinical status (p = 0.0013). The highest seropositivity was observed among mares with a history of abnormal vaginal discharge (68.42%), followed by estrous cycle disturbances (52.94%), dystocia (40.00%), abortion (37.50%), vaginal lesions (33.33%), apparently healthy status (33.33%), and vaginitis (25.00%). No ELISA-positive cases were detected among the two mares classified as infertile (Table 1). These values correspond to the clinical distributions reported in the manuscript.

Among stallions, ELISA seropositivity also differed significantly among the recorded clinical categories (p = 0.0308). Seropositivity was detected in seven apparently healthy stallions (15.56%) and one stallion with genital inflammation (9.09%), whereas no ELISA-positive cases were detected among stallions with abnormal genital discharge or genital lesions (Table 2).

### Association of molecular findings with clinical findings

Among mares, PCR positivity differed significantly according to reproductive and clinical status (p = 0.0012). The highest proportion of PCR-positive animals occurred among mares classified as infertile (50.00%), followed by apparently healthy mares (25.00%), mares with a history of abortion (25.00%), abnormal vaginal discharge (21.05%), vaginal lesions (16.67%), estrous cycle disturbances (11.76%), and dystocia (10.00%) (Table 1).

Among stallions, PCR-positive results were detected only in the apparently healthy group. No significant association was detected between PCR positivity and clinical category (p = 0.0719), and no PCR-positive cases were identified among stallions with genital inflammation, abnormal genital discharge, or genital lesions (Table 2).

**Table 1:** Distribution of ELISA and PCR positivity for Taylorella equigenitalis according to clinical findings in mares (n = 84).

**Clinical finding**	**Total no.**	**ELISA-positive, n (%)**	**PCR-positive, n (%)**
Apparently healthy	36	12 (33.33)	9 (25.00)
Abnormal vaginal discharge (at least once)	19	13 (68.42)**	4 (21.05)
Dystocia (at least once)	10	4 (40.00)	1 (10.00)
Estrous cycle disturbances (at least once)	17	9 (52.94)	2 (11.76)
Abortion (at least once)	8	3 (37.50)	2 (25.00)
Infertility (temporary or permanent)	2	0 (0.00)	1 (50.00)**
Vaginal lesions (at least one lesion, including redness, ulceration, pimples, sloughing, or masses)	6	2 (33.33)	1 (16.67)
Vaginitis (at least once)	4	1 (25.00)	1 (25.00)
p-value		0.0013	0.0012
95% CI		19.63–53.00	12.67–33.45

p* < *0*.*05 (*); p < 0.01 (**)*; *p* < *0.001 (***); p* <* 0.0001 (****)*.* CI = Confidence interval; ELISA = Enzyme-linked immunosorbent assay; PCR = Polymerase chain reaction. Individual horses could contribute to more than one clinical category.

**Table 2:** Distribution of ELISA and PCR positivity for Taylorella equigenitalis according to clinical findings in stallions (n = 73).

**Clinical finding**	**Total no.**	**ELISA-positive, n (%)**	**PCR-positive, n (%)**
Apparently healthy	45	7 (15.56)*	2 (4.44)
Genital inflammation (penis, prepuce, and/or testis)	11	1 (9.09)	0 (0.00)
Abnormal genital discharge	7	0 (0.00)	0 (0.00)
Genital lesions (at least one lesion, including redness, ulceration, pimples, sloughing, or masses)	14	0 (0.00)	0 (0.00)
p-value		0.0308	0.0719
95% CI		5.915–18.24	1.331–2.551

p* < *0*.*05 (*); p < 0.01 (**)*; *p* < *0.001 (***); p* <* 0.0001 (****)*.* CI = Confidence interval; ELISA = Enzyme-linked immunosorbent assay; PCR = Polymerase chain reaction. Individual horses could contribute to more than one clinical category.

### Association of seropositivity with epidemiological factors

ELISA seropositivity differed significantly among age groups (p = 0.0041). Horses aged >10 years had the highest seropositivity at 62.86% (22/35), followed by those aged 7–10 years (33.33%, 17/51), >5–6 years (23.81%, 10/42), and the youngest study group (10.35%, 3/29). The corresponding OR and RR analyses also differed significantly among age groups (both p = 0.0001) (Table 3).

Seropositivity was significantly associated with sex (p = 0.0248), with a higher proportion observed among females (52.38%, 44/84) than males (10.96%, 8/73). The OR and RR analyses also differed significantly according to sex (both p = 0.0001) (Table 3).

Seropositivity also differed significantly among provinces (p = 0.0237). The highest proportion was recorded in Baghdad (39.71%, 27/68), followed by Wasit (36.84%, 14/38), Babylon (30.00%, 9/30), and Nineveh (9.52%, 2/21). The corresponding OR and RR analyses were reported as significantly different among geographic categories (both p = 0.0001) (Table 3). The manuscript reports these geographic distributions consistently.

**Table 3:** Association of Taylorella equigenitalis seropositivity with selected epidemiological factors.

**Factor**	**Positive/total, n**	**Positivity, % (95% CI)**	**OR (95% CI)**	**RR (95% CI)**
Age (years)				
4–5	3/29	10.35 (3.58–26.39)	0.19 (0.05–0.65)	0.27 (0.09–0.81)
>5–6	10/42	23.81 (13.48–38.53)	0.54 (0.24–1.22)	0.65 (0.36–1.18)
7–10	17/51	33.33 (21.97–47.03)	1.01 (0.50–2.06)	1.01 (0.63–1.62)
>10	22/35	62.86** (46.34–76.83)	5.19**** (2.33–11.55)	2.56**** (1.71–3.82)
p-value		0.0041	0.0001	0.0001
Sex				
Male	8/73	10.96 (5.66–20.16)	0.11 (0.05–0.26)	0.21 (0.11–0.42)
Female	44/84	52.38* (41.83–62.72)	8.94**** (3.82–20.91)	4.78**** (2.41–9.84)
p-value		0.0248	0.0001	0.0001
Region				
Baghdad	27/68	39.71 (28.93–51.58)	1.69**** (0.86–3.30)	1.41**** (0.91–2.20)
Babylon	9/30	30.00 (16.66–47.88)	0.84 (0.35–1.98)	0.89 (0.49–1.61)
Nineveh	2/21	9.52 (2.65–28.91)	0.18 (0.04–0.81)	0.26 (0.07–0.99)
Wasit	14/38	36.84 (23.83–52.72)	1.24 (0.58–2.67)	1.15 (0.71–1.89)
p-value		0.0237	0.0001	0.0001

p* < *0*.*05 (*); p < 0.01 (**)*; *p* < *0.001 (***); p* <* 0.0001 (****)*.* CI = Confidence interval; OR = Odds ratio; RR = Relative risk.

### Association of molecular findings with epidemiological factors

PCR positivity differed significantly among age groups (p = 0.0345). The highest PCR positivity was detected among horses aged >5–6 years (38.10%, 16/42), followed by those aged 7–10 years (9.80%, 5/51) and >10 years (5.71%, 2/35); no PCR-positive animals were detected in the youngest study group (0/29). The OR and RR analyses also differed significantly among age categories (both p = 0.0001) (Table 4). The value of 38.10% is supported by 16/42 and by Table 4.

PCR positivity was significantly associated with sex (p = 0.0131), with a higher proportion detected among females (25.00%, 21/84) than males (2.74%, 2/73). The corresponding OR and RR analyses also differed significantly according to sex (both p = 0.0001) (Table 4).

Geographically, PCR positivity was 16.18% (11/68) in Baghdad, 15.79% (6/38) in Wasit, 13.33% (4/30) in Babylon, and 9.52% (2/21) in Nineveh. The manuscript reports an overall difference among provinces at p = 0.029, with the OR and RR analyses reported at p = 0.0001 (Table 4).

**Table 4:** Association of PCR positivity for Taylorella equigenitalis with selected epidemiological factors.

**Factor**	**Positive/total, n**	**Positivity, % (95% CI)**	**OR (95% CI)**	**RR (95% CI)**
Age (years)				
4–5	0/29	0.00 (0–11.70)	0.08 (NA)	0.09 (NA)
>5–6	16/42	38.10* (25.00–53.19)	9.495**** (3.54–25.45)	6.26**** (2.77–14.14)
7–10	5/51	9.80 (4.26–20.98)	0.53 (0.19–1.52)	0.58 (0.23–1.47)
>10	2/35	5.71 (1.58–18.61)	0.29 (0.06–1.31)	0.33 (0.08–1.35)
p-value		0.0345	0.0001	0.0001
Sex				
Male	2/73	2.74 (0.75–9.45)	0.085 (0.02–0.37)	0.11 (0.03–0.45)
Female	21/84	25.00* (16.97–35.21)	11.83**** (2.67–52.48)	9.13**** (2.21–37.60)
p-value		0.0131	0.0001	0.0001
Region				
Baghdad	11/68	16.18* (9.28–26.69)	1.24**** (0.51–3.01)	1.20**** (0.56–2.55)
Babylon	4/30	13.33* (5.31–29.68)	0.87 (0.27–2.79)	0.89 (0.33–2.43)
Nineveh	2/21	9.52 (2.65–28.91)	0.58 (0.12–2.66)	0.62 (0.16–2.44)
Wasit	6/38	15.79* (7.44–30.42)	1.13 (0.41–3.09)	1.11 (0.47–2.60)
p-value		0.029	0.0001	0.0001

p* < *0*.*05 (*); p < 0.01 (**)*; *p* < *0.001 (***); p* <* 0.0001 (****)*.* CI = Confidence interval; NA = Not available; OR = Odds ratio; PCR = Polymerase chain reaction; RR = Relative risk.

### Phylogenetic and genetic diversity analyses

Sequences obtained from the 23 PCR-positive samples, designated GhIQf-Equine 1–GhIQf-Equine 23, were deposited in the NCBI GenBank database under accession numbers PZ146151.1–PZ146173.1. Phylogenetic relationships among the Iraqi *T. equigenitalis* sequences and reference sequences retrieved from GenBank were inferred from partial *16S rRNA* gene sequences using the maximum-likelihood method. Branch lengths represented estimated evolutionary divergence, and the scale bar corresponded to 0.01 substitutions per site. Bootstrap support values were displayed at the corresponding internal nodes, with several major nodes showing support of 99%–100%.

Pairwise sequence comparison showed that the Iraqi sequences had 97.18%–99.12% nucleotide identity with the Iranian *T. equigenitalis* isolate Tay.Zahraei1 (GenBank accession No. DQ840456.1) (Table 5). GhIQf-Equine 10 showed the highest identity (99.12%), whereas GhIQf-Equine 2 and GhIQf-Equine 20 showed the lowest identity (97.18%). The phylogenetic tree did not contain a taxonomically distinct outgroup; sequences positioned toward the basal portion of the topology, including IR-CEM/Iran, KITE-1/Korea, HH139/Japan, 96-178/USA, and Kentucky 188/Japan, were also identified as *T. equigenitalis* (Figure 6). The manuscript reports the overall identity range and lack of a distinct outgroup accordingly.

**Table 5:** Pairwise nucleotide identity between Iraqi Taylorella equigenitalis sequences and the Iranian Tay.Zahraei1 isolate (GenBank accession No. DQ840456.1).

**Isolate**	**Accession No.**	**Aligned identity, bp**	**Identity (%)**	**Query cover (%)**	**Similarity**
GhIQf-Equine 1	PZ146151.1	558/568	98.24	100	Very high
GhIQf-Equine 2	PZ146152.1	552/568	97.18	99	High
GhIQf-Equine 3	PZ146153.1	556/568	97.89	99	High
GhIQf-Equine 4	PZ146154.1	554/568	97.54	99	High
GhIQf-Equine 5	PZ146155.1	561/568	98.77	99	Very high
GhIQf-Equine 6	PZ146156.1	556/568	97.89	99	High
GhIQf-Equine 7	PZ146157.1	560/568	98.59	98	Very high
GhIQf-Equine 8	PZ146158.1	556/568	97.89	98	High
GhIQf-Equine 9	PZ146159.1	554/568	97.54	99	High
GhIQf-Equine 10	PZ146160.1	563/568	99.12	99	Highest
GhIQf-Equine 11	PZ146161.1	557/568	98.06	99	Very high
GhIQf-Equine 12	PZ146162.1	555/568	97.71	99	High
GhIQf-Equine 13	PZ146163.1	558/568	98.24	99	Very high
GhIQf-Equine 14	PZ146164.1	554/568	97.54	97	High
GhIQf-Equine 15	PZ146165.1	555/568	97.71	98	High
GhIQf-Equine 16	PZ146166.1	556/568	97.89	98	High
GhIQf-Equine 17	PZ146167.1	558/568	98.24	98	Very high
GhIQf-Equine 18	PZ146168.1	557/568	98.06	98	Very high
GhIQf-Equine 19	PZ146169.1	558/568	98.24	98	Very high
GhIQf-Equine 20	PZ146170.1	552/568	97.18	98	High
GhIQf-Equine 21	PZ146171.1	556/568	97.89	98	High
GhIQf-Equine 22	PZ146172.1	557/568	98.06	98	Very high
GhIQf-Equine 23	PZ146173.1	554/568	97.54	97	High

Genetic diversity analysis identified 521 conserved sites, 32 variable sites, 32 polymorphic sites, 12 parsimony-informative sites, and 20 singleton variable sites. Thirteen haplotypes were detected, with a haplotype diversity of 0.909 and nucleotide diversity (π) of 0.0105. The average number of nucleotide differences was 5.81, and the overall mean genetic distance was 0.0105. Mean genetic distances were 0.0046 within the Iraqi sequences, 0.0204 within the reference sequences, and 0.0202 between the Iraqi and reference groups. Tajima’s D was −1.0503 (Table 6).

**Table 6:** Genetic diversity analysis of the Iraqi and reference Taylorella equigenitalis sequences.

**Marker**	**Value**
Conserved sites	521 bp
Variable sites	32 bp
Polymorphic sites	32 bp
Parsimony-informative sites	12 bp
Singleton variable sites	20 bp
Number of haplotypes	13
Haplotype diversity	0.909
Nucleotide diversity (π)	0.0105
Average number of nucleotide differences	5.81
Mean genetic distance	0.0105
Within Iraqi isolates	0.0046
Within reference strains	0.0204
Between Iraqi and reference groups	0.0202
Tajima’s D	−1.0503

NCBI = National Center for Biotechnology Information.

**Figure 6:**
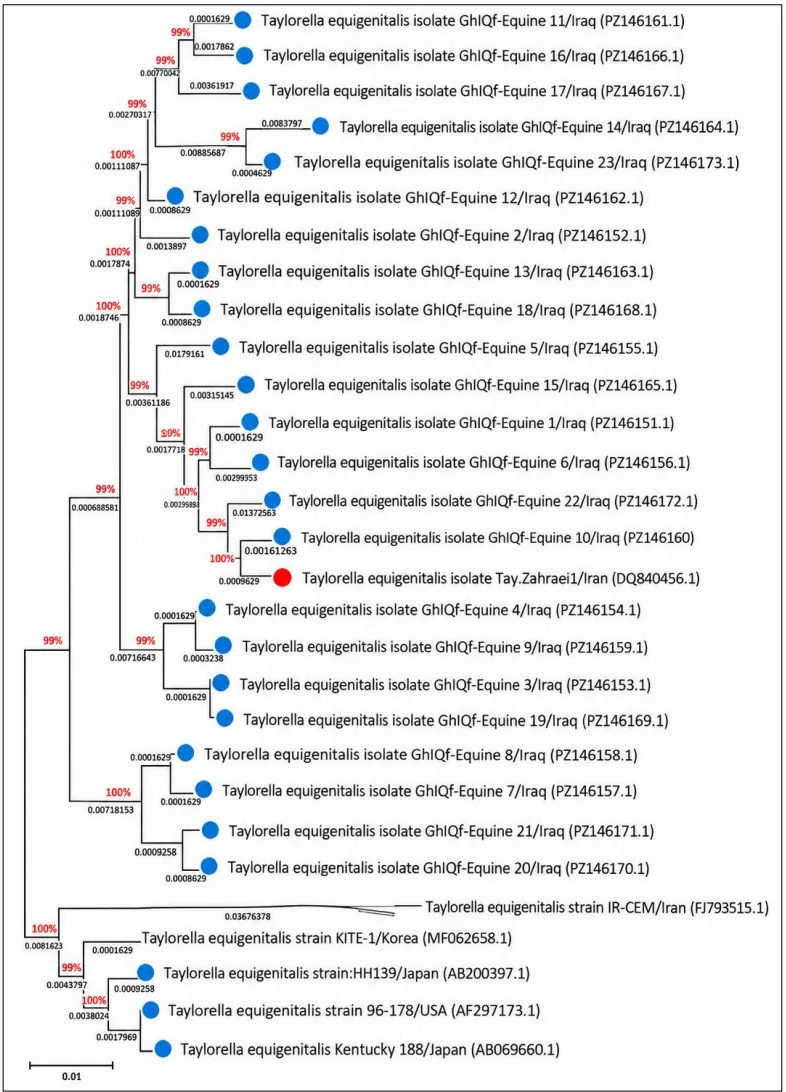
Maximum-likelihood phylogenetic tree analysis based on partial *16S rRNA* gene sequence showing relationship between local *Taylorella* *equigenitalis* isolates (blue circle), global identical isolate (red circle), and other NCBI BLAST isolates/strains.

## DISCUSSION

### Serological and molecular detection of *T. equigenitalis*

The present study demonstrated serological and molecular evidence of *T. equigenitalis* among horses in Iraq, with 33.12% of the examined horses testing positive by ELISA and 14.65% testing positive by PCR. The difference between serological and molecular positivity highlights the distinct biological information provided by the two diagnostic approaches. Previous studies have reported considerable variation in the detection of *T. equigenitalis* among horse populations. Parlevliet *et al.* [22] reported PCR positivity in 54 (50.47%) of 107 asymptomatic mares in the Netherlands, including 14/36 (38.89%) PCR-positive horses of Icelandic origin. In Slovenia, Zdovc *et al.* [23] examined 980 genital swabs collected from 245 stallions and detected 17 (6.94%) culture-positive stallions, with three additional animals detected by PCR. In a small Croatian survey involving 12 horses, culture results were negative, whereas PCR detected *Taylorella* spp. in three (25%) horses; subsequent sequencing confirmed *T. equigenitalis* in one horse [24].

Considerably lower occurrence has been reported in some large-scale surveillance programs. A South African national screening program using quantitative PCR (qPCR) with culture confirmation detected *T. equigenitalis* in 39/3820 (1.02%) horses, comprising 36/3703 (0.97%) stallions and 3/117 (2.56%) mares distributed across 12 properties in three provinces [8]. In contrast, surveillance of Thoroughbred horses in Korea detected *T. equigenitalis* by qPCR in 71/526 (13.5%) horses [14]. In a Swedish retrospective investigation, Al-Kass *et al.* [25] reported *T. equigenitalis* in 53/25,512 (0.21%) examined samples, including 11/2308 (0.48%) stallions. In Iran, Aalinasab *et al.* [9] detected *T. equigenitalis* in 4/240 (1.70%) infertile mares. More recently, Solbach *et al.* [7] detected the bacterium by qPCR in 52/361 (14.40%) Icelandic horses located in southern Germany and Austria. Differences between these studies and the present findings may reflect variation in study populations, sampling strategies, diagnostic methods, anatomical sampling sites, sample handling, geographic setting, management practices, and study periods. Direct comparison should therefore be interpreted cautiously because the cited investigations differed substantially in design and diagnostic approach.

### Agreement and discordance between ELISA and PCR

The present study identified different positivity rates using ELISA and PCR. Among the 157 horses, 21 (13.38%) were positive by both methods, 31 (19.74%) were ELISA-positive/PCR-negative, two (1.27%) were ELISA-negative/PCR-positive, and 103 (65.61%) were negative by both methods. Thus, most PCR-positive horses were also seropositive, whereas a substantially larger group had detectable antibodies without simultaneous PCR detection.

This discordance should be interpreted in the context of the different targets of the assays. ELISA detects antibodies and may therefore identify previous or ongoing exposure, whereas PCR detects bacterial DNA in the sampled material at the time of collection. Serological findings can be influenced by the timing and magnitude of the antibody response, cross-reactivity, nonspecific antibody binding, antigen characteristics, cutoff selection, and individual immunological variation [26, 27]. Conversely, PCR-negative results in exposed or infected animals may occur when the bacterial load is low, shedding is intermittent, the selected sampling site does not contain detectable organisms, sampling is suboptimal, DNA deteriorates during processing, or amplification is affected by inhibitors present in clinical specimens [28, 29]. Consequently, the higher ELISA positivity observed in this study should not be interpreted as evidence of lower PCR performance or reduced ELISA specificity without direct diagnostic accuracy evaluation against an appropriate reference standard.

### Clinical associations and reproductive disorders

Clinical findings demonstrated a significant association between serological and molecular positivity and reproductive status in mares, whereas PCR positivity was not significantly associated with the recorded clinical categories in stallions. Among mares, the highest ELISA positivity occurred in those with a history of abnormal vaginal discharge (68.42%), whereas the highest PCR positivity occurred among mares classified as infertile (50%). In stallions, PCR-positive results were detected only among apparently healthy animals, supporting the epidemiological importance of clinically inapparent carriage. Similar differences in the clinical expression of *T. equigenitalis* infection between mares and stallions have been described previously [6, 9, 13, 22, 30–33].

The reproductive abnormalities detected among serologically or molecularly positive mares are biologically compatible with the known involvement of *T. equigenitalis* in the female reproductive tract. Infection-associated inflammatory changes within the endometrium and reproductive tract may affect uterine function and reproductive performance [20, 34]. Previous studies have similarly described transient reproductive dysfunction in naturally infected mares [35, 36]. In contrast, stallions may harbor *T. equigenitalis* without overt clinical manifestations, thereby complicating recognition and control of infection [12, 37, 38]. The presence of clinically inapparent carriers is particularly relevant to CEM surveillance because apparently healthy breeding animals may contribute to maintenance and dissemination of the organism. International movement and trade of horses have also contributed historically to the geographic dissemination of CEM and underscore the importance of appropriate surveillance and biosecurity measures [39].

Nevertheless, the clinical associations observed in the present study should not be interpreted as proof that *T. equigenitalis* caused each recorded reproductive disorder. The cross-sectional design identifies associations between diagnostic positivity and clinical history but does not establish temporal or causal relationships.

### Associations with age and sex

Age was significantly associated with both serological and molecular findings, although the patterns differed between diagnostic methods. Seropositivity was highest among horses aged >10 years (62.86%), whereas PCR positivity was highest among horses aged >5–6 years (38.10%). The higher seropositivity among older horses may reflect cumulative exposure over successive breeding seasons and greater lifetime opportunities for contact with infected animals. Repeated breeding activity may similarly increase the probability of previous exposure or prolonged colonization [11, 40]. Because ELISA detects antibodies rather than the organism itself, accumulation of previous exposure with increasing age offers a plausible explanation for the observed serological pattern.

In contrast, the highest molecular positivity in the present study occurred among horses aged 5–6 years. This finding may reflect differences in current exposure or bacterial detection across age groups; however, the study did not directly measure breeding frequency, mating activity, duration of colonization, or bacterial shedding. Therefore, specific behavioral or reproductive mechanisms underlying the age-associated PCR pattern cannot be established from the available data. Solbach *et al.* [7] similarly reported that advancing age was not associated with greater *T. equigenitalis* positivity, indicating that age-related patterns may vary among populations and epidemiological settings.

Sex was also significantly associated with both ELISA and PCR positivity. Females had substantially higher seropositivity (52.38%) than males (10.96%) and higher PCR positivity (25.00%) than males (2.74%). Higher positivity among mares has also been reported in other investigations [6, 14]. This finding is consistent with the more evident reproductive manifestations that may occur after colonization of the female reproductive tract. Nevertheless, the lower positivity detected among stallions in the present study does not diminish their potential epidemiological importance. Previous studies have demonstrated that stallions may harbor *T. equigenitalis* on external genital sites for prolonged periods while remaining clinically unaffected and can consequently contribute to maintenance and venereal transmission of CEM [7, 30].

### Geographic variation

Serological and molecular positivity varied among the four study provinces. Seropositivity was highest in Baghdad (39.71%) and Wasit (36.84%), followed by Babylon (30.00%) and Nineveh (9.52%). PCR positivity showed a narrower geographic range, with positivity of 16.18% in Baghdad, 15.79% in Wasit, 13.33% in Babylon, and 9.52% in Nineveh. These geographic differences may reflect variation in the populations sampled and local epidemiological conditions.

Differences in horse population structure, breeding management, animal movement, frequency of contact among breeding animals, and access to veterinary supervision have been proposed as factors that can influence transmission of equine infectious diseases [41–43]. However, these variables were not directly quantified in the present study. Therefore, the observed provincial differences should be regarded as epidemiological associations rather than evidence that any specific management or movement factor was responsible for the geographic distribution of *T. equigenitalis*.

### Phylogenetic relationships and genetic diversity

Molecular characterization of the 23 Iraqi *T. equigenitalis* sequences demonstrated measurable genetic variation within the analyzed partial *16S rRNA* gene region. Genetic diversity analysis identified 13 haplotypes, with a haplotype diversity of 0.909 and nucleotide diversity (π) of 0.0105. The mean genetic distance within the Iraqi sequences was 0.0046, compared with 0.0204 among the reference sequences and 0.0202 between the Iraqi and reference groups. These findings indicate that multiple sequence variants were represented among the PCR-positive Iraqi samples while overall nucleotide divergence within the Iraqi group remained relatively limited.

Sequence comparisons also demonstrated 97.18%–99.12% nucleotide identity between the Iraqi sequences and the Iranian Tay.Zahraei1 *T. equigenitalis* isolate (GenBank accession No. DQ840456.1), with GhIQf-Equine 10 showing the highest identity (99.12%). The high sequence similarity demonstrates genetic relatedness within the analyzed *16S rRNA* gene region. Previous molecular studies have documented genetic variation and closely related lineages among *T. equigenitalis* isolates obtained from different geographic locations [21, 44, 45].

The presence of 32 polymorphic sites, including 12 parsimony-informative and 20 singleton variable sites, further demonstrates sequence variation within the analyzed dataset. However, the partial *16S rRNA* sequence data used in the present study do not provide sufficient resolution to establish specific transmission lineages, recent clonal expansion, direction of transmission, common geographic origin, or direct epidemiological linkage between Iraqi and Iranian isolates. Therefore, the observed sequence similarity should be interpreted as molecular relatedness rather than evidence of cross-border transmission. Higher-resolution approaches, including multilocus sequence typing or whole-genome sequencing combined with detailed animal movement and breeding histories, would be required to resolve these epidemiological relationships more precisely [21, 44, 45].

The negative Tajima’s D value (−1.0503) was also observed in the analyzed dataset. However, because statistical significance for Tajima’s D was not reported and the analysis was based on partial *16S rRNA* sequences, this finding alone does not establish recent population expansion, purifying selection, or another specific evolutionary process. Further genomic investigation with a larger and epidemiologically representative collection of isolates would be required to characterize the population structure and evolutionary dynamics of *T. equigenitalis* circulating among horses in Iraq.

## CONCLUSION

The present study provides integrated serological, molecular, epidemiological, and phylogenetic evidence of *T. equigenitalis* among horses examined in Iraq. Of the 157 horses, 52 (33.12%) were seropositive by ELISA and 23 (14.65%) were PCR-positive, with 21 (13.38%) positive by both methods. Seropositivity was highest among mares with abnormal vaginal discharge (68.42%), horses aged >10 years (62.86%), and females (52.38%), whereas PCR positivity was highest among mares classified as infertile (50.00%), horses aged >5–6 years (38.10%), and females (25.00%). Geographic differences were also observed, with the highest seropositivity in Baghdad and Wasit and the highest PCR positivity in Baghdad. Sequencing of the 23 PCR-positive samples generated GenBank accession numbers PZ146151.1–PZ146173.1, and the Iraqi sequences showed 97.18%–99.12% nucleotide identity with the Iranian *T. equigenitalis* isolate DQ840456.1. Genetic analysis further identified 13 haplotypes, a haplotype diversity of 0.909, and nucleotide diversity of 0.0105. These principal findings are consistent with the quantitative results reported throughout the manuscript.

From a practical perspective, the coexistence of seropositive and PCR-positive horses, together with the detection of PCR positivity among apparently healthy stallions, emphasizes the need to include clinically inapparent animals in CEM surveillance. Reliance on clinical examination alone may fail to identify potential carriers. The complementary use of serological and molecular testing may therefore provide broader epidemiological information by identifying previous or current exposure through antibody detection and detecting bacterial DNA in genital samples. These findings support consideration of routine screening of breeding horses, particularly before mating, movement, or introduction into breeding populations, alongside appropriate biosecurity and reproductive health monitoring.

A major strength of the study is the combined application of clinical assessment, ELISA, PCR, sequencing, phylogenetic analysis, and epidemiological evaluation within the same horse population. Sampling across Baghdad, Babylon, Nineveh, and Wasit also provided data from geographically distinct areas, while deposition of the 23 PCR-positive sequences in GenBank enables future molecular comparison with isolates identified elsewhere. The study therefore establishes useful baseline data for subsequent surveillance and molecular epidemiological investigations of *T. equigenitalis* in Iraq.

Several limitations should, however, be considered when interpreting the findings. The cross-sectional design does not establish temporal or causal relationships between *T. equigenitalis* positivity and reproductive disorders or other epidemiological characteristics. The sampled horses were obtained from selected provinces and were not described as a nationally representative random sample; therefore, the observed positivity rates should not be generalized directly to the entire Iraqi horse population. ELISA and PCR measure different biological targets, and the study did not evaluate their diagnostic sensitivity or specificity against an independent reference standard. In addition, single-time-point genital sampling may not detect animals with low bacterial loads or intermittent shedding. The phylogenetic analysis was based on partial *16S rRNA* gene sequences, which provides limited resolution for distinguishing closely related strains, reconstructing transmission chains, or demonstrating direct epidemiological connections between Iraqi and geographically neighboring isolates. Finally, several clinical categories contained relatively small numbers of animals, which may limit the precision of some subgroup comparisons.

Future studies should include larger, prospectively designed and geographically representative horse populations encompassing additional Iraqi provinces and breeding systems. Repeated sampling of individual horses would help clarify persistence, intermittent shedding, and changes in diagnostic status over time. Studies incorporating culture together with validated molecular and serological methods would permit more rigorous assessment of diagnostic concordance and performance. Detailed information on breeding history, animal movement, mating practices, importation, management, and contact networks should also be incorporated to identify determinants of transmission more reliably. Higher-resolution molecular approaches, including multilocus sequence typing and whole-genome sequencing, combined with epidemiological metadata, are particularly warranted to characterize strain diversity, determine whether transmission clusters occur within Iraq, and evaluate possible regional relationships with isolates reported from neighboring countries.

Overall, the findings demonstrate that *T. equigenitalis* is detectable serologically and molecularly among horses in the examined Iraqi populations and is associated with reproductive and epidemiological characteristics. The study provides an initial molecular framework for CEM surveillance in Iraq but does not establish national prevalence or specific transmission pathways. Continued surveillance, routine screening of breeding horses, strengthened diagnostic capacity, improved biosecurity, and higher-resolution molecular epidemiological studies are warranted to better define transmission dynamics and support evidence-based prevention and control of CEM in the Iraqi equine population.

## DATA AVAILABILITY

All data are included in the manuscript.

## GENERATIVE AI DECLARATION

The authors declare that no generative artificial intelligence or AI-assisted technologies were used in the writing, analysis, or preparation of this manuscript. 

## AUTHORS’ CONTRIBUTIONS

ZSH: Study design, clinical examination, and preparation of the original draft. AMR: Molecular processing and analysis of the study samples. STM: Blood sample collection and serological examination. HAJG: Statistical analysis, submission of sequence data to NCBI GenBank, and phylogenetic analysis. All authors reviewed and edited the manuscript and approved the final version.
